# Evaluation of Protocols of Controlled Ovarian Stimulation in Obtaining Mature Oocytes (MII): Retrospective Study on Assisted Reproductive Technology Procedures

**DOI:** 10.5935/1518-0557.20210077

**Published:** 2022

**Authors:** Cátia Manuela Ribeiro Barbosa Martins, Patrícia Cordeiro Pires de Figueiredo Gomes Crisóstomo Ruivo, Denise Cristina Mós Vaz-Oliani, Renato Alessandre Silva Martins, Antonio Helio Oliani

**Affiliations:** 1 University of Beira Interior - Faculty of Health Sciences, Covilhã, Portugal; 2 Reproductive Medicine Unit - University Hospital Center Cova da Beira, Covilhã, Portugal; 3 Institute of Reproductive and Fetal Medicine - Faculty of Medicine of São José do Rio Preto, São José do Rio Preto, Brazil

**Keywords:** infertility, assisted reproductive technology, anti-Müllerian hormone, antral follicle count, controlled ovarian stimulation

## Abstract

**Objective:**

To understand which of the controlled ovarian stimulation (COS) protocols used in different patients are associated with greater amounts of oocytes retrieved.

**Methods:**

The study population was divided into three groups, considering AMH and AFC to obtain the Ovarian Response Predictor Index (ORPI); they were grouped into: G1-Low Reserve (ORPI <0.5); G2-Normal Reserve (ORPI:0.5-0.9); and G3-High Reserve (ORPI≥0.9). 246 cycles were selected in which COS was used: recombinant FSH - follitropin alfa or beta (Protocol 1) or corifollitropin alfa (Protocol 2), both associated with urinary HMG and the GnRH antagonist, with the trigger performed using recombinant hCG or GnRH agonist.

**Results:**

The number of oocytes obtained was higher in protocol 1 in all groups, with higher counts seen in G1 than in G2 or G3. The number of days required in COS for protocol 2 was greater than for protocol 1 in all groups. The total dose of recombinant FSH alfa or beta / urinary HMG used in protocol 1 was inversely proportional to the ovarian reserve. The lower the ORPI, the greater the average number of international units administered. In protocol 2, there was a need to supplement with higher doses of urinary HMG when compared to protocol 1. The dosage of the GnRH antagonist was dependent on the number of COS days until the trigger was used. In obtaining MII oocytes, the percentages were similar regardless of the trigger used.

**Conclusions:**

The use of follitropin leads to greater numbers of retrieved oocytes than corifollitropin alfa in all ORPIs. The dose of recombinant FSH used with urinary HMG increases inversely proportional to the ORPI value. The fixed dose of recombinant FSH deposit requires a sharp increase in the dose of urinary HMG.

## INTRODUCTION

The World Health Organization (WHO) has considered infertility a Public Health problem. It has been defined as "a disease of the reproductive system translated into the inability to obtain a pregnancy after 12 months or more of regular sexual intercourse and without using contraception" (Direcção Geral de Saúde, 2010; Vander Borght & Wyns, 2018).

The prevalence of infertility is exceedingly difficult to assess, with studies reporting the possibility that one in four women may have difficulties in becoming pregnant throughout their lives (Direcção Geral de Saúde, 2010). Recent studies report a prevalence of infertility that varies between 3.5% and 16.7% in the most developed countries and 6.9% to 9.3% in the least developed countries (Direcção Geral de Saúde, 2010; Vander Borght & Wyns, 2018)

In view of these data, and since the prevalence of infertility has grown globally, couples are more actively seeking ​​reproductive health clinics (Gnoth *et al.,* 2005). In Portugal, there has been an increase in activity in this area. However, this increase is still insufficient to respond to the problems of couples with indication for Assisted Reproductive Technology (ART) treatments.

Evidence suggests that the prevalence of infertility continues to increase. The causes for such increase include the decision by couples of having children at a later age, increased obesity, consumption of tobacco, alcohol, and other harmful substances, and the increasing exposure of couples to high levels of stress. (Direcção Geral de Saúde, 2010; Vander Borght & Wyns, 2018; Deshpande & Gupta, 2019)

The relationship between a woman's age and fertility is already well known; the older the age, the lower the quantity and quality of oocytes (Vollenhoven & Hunt, 2018; Liu & Case, 2011). At birth, the human ovary contains one to two million primordial quiescent follicles. Only a small part of these (between 300 and 400,000) will mature during a woman's reproductive life. Nuclear maturation of oocytes, accompanied by cytoplasmic maturation, shows their evolution from the prophase I stage, during recruitment and follicular development, to metaphase II (stage MII), which occurs in final maturation and subsequent ovulation (Vollenhoven & Hunt, 2018; Liu & Case, 2011; Braga *et al.,* 2020).

The ovarian reserve describes the functional potential of the ovary, reflecting the quantity and possible quality of oocytes. The antral follicles count (AFC) is defined as the number of follicles with a diameter of less than 10 mm evaluated in the early follicular phase. It is a good marker of ovarian reserve obtained via endovaginal ultrasound examination of the ovaries (Silva & Vilodre, 2009; Amanvermez & Tosun, 2016).

The anti-Müllerian hormone (AMH) is secreted by the granulosa cells of the pre-antral and growing antral follicles. AMH production occurs during follicle differentiation from the primordial to the primary phase and continues until the follicles reach stages with diameters from 2 to 6mm. Some authors describe AMH values as low when they are < 0.5ng/mL; intermediate when they range between 1.0 and 3.5ng/mL; and high when they are > 3.5ng/ mL. Although it is important to estimate the ovarian reserve, AMH cannot be used to assess oocyte or embryo quality. Compared to AFC, AMH has the advantage of showing little variation over the menstrual cycle and between cycles. (Dewailly & Laven, 2019; Usta & Oral, 2012; La Marca *et al*., 2010).

AFC, AMH, and female age are important parameters to predict response to controlled ovarian stimulation (COS) and identify women who are unresponsive to treatment, at risk of canceling the cycle, and hyper-responsive women at higher risk of developing Ovarian Hyperstimulation Syndrome (OHSS) (Oliani *et al.,* 2019).

The Ovarian Response Prediction Index (ORPI) is calculated using the formula below:


ORPI=AMHxAFC/Womanage

The ORPI may aid in the choice of type of COS and help predict patient response to the proposed treatment based to her index, as follows: poor response is expected in women with an ORPI <0.5; women with an ORPI of [0.5-0.9] are expected to present optimal response to treatment; and women with an ORPI ≥ 0.9 are expected to present hyper-response to treatment (Oliani *et al*., 2019; Oliveira *et al*., 2012). With COS, the goal is to obtain an adequate number of quality oocytes for each treatment cycle. 

In vivo, oocyte maturation occurs because of natural selection. In contrast, in ART cycles, growth and maturation of multiple follicles is induced using supra-physiological doses of gonadotropins, to make sure a desired number of mature oocytes is obtained (Braga *et al.,* 2020; Hodgen, 1989; Quaas & Legro, 2019). Different COS protocols entail different combinations of hormones that may lead to follicular asynchrony and variations in the quantity, quality, viability, and competence of oocytes. Therefore, it is extremely important to analyze the quantity and quality of stimulation given to patients (Braga *et al.,* 2020; Quaas & Legro, 2019)

It is important that patients undergo preparation before the initiation of COS. To facilitate scheduling for women undergoing treatment and ART centers, combined oral contraceptives (COC) have been prescribed prior to the initiation of treatment cycles to schedule and date menstruation. According to studies (Biljan *et al*., 1998; Griesinger *et al*., 2008), the use of COC provides other advantages, such as improved follicular timing during ovulation stimulation, lower cancellation rates in hyper-responsive women, and greater number of aspirated oocytes.

Gonadotropins are essential in COS protocols. Follicle-Stimulating Hormone (FSH) is the main hormone in ovulation induction. Various preparations contain FSH and can be used in ovulation stimulation cycles for ART (Oliani *et al*., 2019). The three most used forms are: (1) Urinary Human Menopausal Gonadotropin (uHMG), which contains FSH and Luteinizing Hormone (LH) of urinary origin and is extracted from the urine of menopausal women (Deeks, 2018); (2) highly purified urinary FSH, which contains predominantly urinary FSH (uFSH) and is also extracted from the urine of menopausal women, although it undergoes a process of greater purification from which urinary LH (uLH) (Oliani *et al*., 2019) and Recombinant FSH (rFSH) are extracted, the latter of which contains only FSH (alfa or beta follitropins); it is produced in a laboratory setting and offers unlimited production potential, unlike uHMG and uFSH. Currently, there is also a recombinant form of prolonged release FSH, corifollitropin alfa (Oliani *et al*., 2019; Rombauts & Talmor, 2012).

Corifollitropin alfa is a recombinant gonadotropin with pharmacodynamic properties similar to conventional rFSH, but with distinct pharmacokinetic characteristics conferring it clinically relevant practical advantages. A single injection of corifollitropin alfa can initiate and maintain multiple follicular developments during the first seven days of ovarian stimulation prior to Ovum Pick Up (OPU) and In Vitro Fertilization (IVF) or Intracytoplasmic Sperm Injection (ICSI), thus requiring fewer drug interventions (Ledger *et al.,* 2011). In contrast with the use of daily FSH preparations, dose adjustments with corifollitropin alfa are not possible during the first seven days of ovarian stimulation, since corifollitropin alfa remains pharmacologically active during this period (Ledger *et al.,* 2011).

In ART cycles, Gonadotropin-Releasing Hormone (GnRH) analogs are also used. They are not ovulation inducers, but endocrine modulators used during ovulation stimulation to avoid the spontaneous early peak of LH, responsible for ovulation and follicular luteinization (Oliani *et al*., 2019). Natural GnRH is a protein, produced by the hypothalamus, with pulsatile release, which has good affinity for its pituitary receptor and a short half-life. GnRH stimulates the pituitary to release FSH and LH. The synthesized GnRH analogs (antagonists and agonists) have a longer half-life than natural GnRH and a greater affinity for pituitary receptors (Oliani *et al*., 2019; Newton *et al*., 2018). Antagonists act by competitive inhibition with natural GnRH in the occupation of receptors, thus creating an immediate pituitary block, with effects observed within a few hours, suppressing the release of FSH and LH. Suppression is more important for LH than for FSH (Oliani *et al*., 2019; Newton *et al*., 2018). GnRH agonists rapidly stimulate the pituitary to release FSH and LH. With the continuous occupation of the same receptors, there is a progressive desensitization over 14 days, with consequent initial elevation and subsequent suppression of FSH and LH secretion (Oliani *et al*., 2019; Newton *et al*., 2018).

GnRH analogues prevent a premature LH surge and avoid the loss of the cycle resulting from early ovulation. Several studies have shown that GnRH antagonists are more potent suppressors than agonists (Oliani *et al*., 2019). The LH peak at the end of folliculogenesis plays an important role in activating oocyte meiosis, with the release, after 36 hours, of a mature oocyte (MII) capable of being fertilized. In COS cycles, when the pituitary is blocked (by GnRH agonists or antagonists), there is no spontaneous LH peak; therefore, the peak must be stimulated using a trigger. The ideal time to use the trigger is when follicles between 18 and 20 mm are viewed in the ultrasound examination performed during COS (Oliani *et al*., 2019).

Human Chorionic Gonadotropin (hCG) can be used as a trigger; in cases in which an antagonist is used in pituitary block, a GnRH agonist should be chosen (Alyasin *et al*., 2016; Le *et al*., 2019). hCG is a gonadotrophic glycoprotein hormone, produced mainly by placental trophoblasts, as the LH produced by the pituitary gland and used in the final maturation of oocytes. hCG can be of urinary origin (uhCG) or recombinant (rhCG), the latter being the one the most used in COS (Youssef *et al*., 2016). GnRH agonists, in addition to being used to block the hypothalamic-pituitary axis, are also used as a trigger for the endogenous release of LH and for the final maturation of oocytes, in cycles in which a GnRH antagonist was used and to prevent complications of ovarian hyperstimulation (Dosouto *et al*., 2017).

Patients with OHSS, a condition currently considered as an iatrogenic syndrome, have multiple follicles and develop ascites, hematological changes, pleural effusion, liver or coagulation disorders which can, in rare cases, be severe or even fatal (Blumenfeld, 2018) OHSS stems from excessive response to hormones used in ovarian stimulation. It is the most feared complication in ART, due to its severity. In the past decade, the replacement of hCG by GnRH agonists in women at risk of hyperstimulation has decreased the incidence and severity of OHSS (Blumenfeld, 2018). The use of GnRH agonists to trigger ovulation is the best option to prevent OHSS and maintain acceptable reproductive results (Blumenfeld, 2018).

The several hormones currently available for COS indicate that there has been a continuous search for a protocol that ofers the best hormonal combination and provides good follicular response in the safest, most efficient, and physiological way possible. However, it has not yet been possible to conclude which is the best protocol.

### Study objectives

The objective of this study was to understand which of the COS protocols used in different patients was associated with greater numbers of oocytes retrieved during OPU, taking into account the number of days of stimulation, the doses of hormones administered, and which trigger obtained greater numbers of mature oocytes (stage MII) for fertilization. Other factors considered were patient age, AFC, and AMH levels, since they are correlated with ORPI and may directly or indirectly influence the success of ART treatments.

Based on the objectives of this study, the following main hypotheses were formulated: there is a significant difference between protocols in the number of oocytes obtained; there is a significant difference between protocols in the length of stimulation; there is a significant difference between protocols in the required stimulation dose; and there is a significant difference between triggers in the percentage of mature oocytes obtained.

## MATERIALS AND METHODS

### Type of study

This retrospective observational study was carried out based on the analysis of clinical processes of patients submitted to ART (exclusively IVF and ICSI) at the Reproductive Medicine Unit of the Hospital and University Center of Cova da Beira (CHUCB) between January 1, 2015 and December 31, 2019.

### Data collection

The data of the patients included in this study were obtained from the clinical files saved in the archives of the Reproductive Medicine Unit at CHUCB. Data collection was carried out in September 2020, after a favorable opinion from the Health Ethics Committee.The data collected from each process were patient age, AFC, AMH level, drugs used in COS, stimulation dosage and length, used trigger, total number of oocytes obtained, and number of mature oocytes only. The data were collected without alterations by the researcher.

### Study participants, inclusion and exclusion criteria, and sample matching

The study population included female patients who underwent IVF or ICSI at the Reproductive Medicine Unit of CHUCB between January 1, 2015 and December 31, 2019. A total of 212 women and 317 COS cycles were included. Of the 317 COS cycles performed in the five years of the study, we selected the cycles in which one of the following protocols were used:

Protocol 1 (203 cycles): rFSH alfa and beta (Gonal^®^/Benfola^®^/Puregon^®^) + uHMG (Menopur^®^) + GnRH antagonist (Orgalutran^®^/Cetrotide^®^); and Protocol 2 (43 cycles): rFSH corifollitropin alfa (Elonva^®^) + uHMG (Menopur^®^) + GnRH antagonist (Orgalutran^®^/ Cetrotide^®^). We decided to select these protocols because they amounted to a representative sample. A total of 246 COS cycles performed in 179 women were included; 124 women were submitted to only one COS cycle, 45 were submitted to two cycles, and 10 underwent three cycles. Female patient age varied between 19 and 39 years, with an average age of 34.51 years. The study included women with all the data required for the study of ovarian reserve, namely: Age, Anti-Müllerian Hormone level, and Antral Follicles count; individuals prescribed COS Protocol 1 or 2 and subjects undergoing IVF or ICSI. Patients with missing information for purposes of this study, individuals on regimans other than Protocols 1 and 2 described above, and women submitted to IVF or ICSI with previously frozen oocytes were excluded. For sample matching, the COS cycles were divided into three groups (G1, G2 and G3) according to ORPI and consequent Ovarian Reserve. G1 included subjects with an ORPI < 0.5, rated as having low ovarian reserve; G2 included subjects with an ORPI of [0.5-0.9], rated as having normal ovarian reserve; and G3 included women with an ORPI ≥0.9, rated as having a high ovarian reserve.

### Statistical methods and procedures

IBM^®^ SPSS Statistics 21^®^ (Statistical Package for the Social Sciences, Inc., Chicago, IL) was used for data treatment and analysis. The frequencies (absolute and relative) of categorical variables and descriptive statistics of continuous variables were analyzed. In order to compare the number of oocytes obtained and the number of days of stimulation (dependent variables) with the Protocol (independent variable or factor), and to compare the proportion of mature oocytes (dependent variable) with the trigger (independent variable), we used the parametric Student's t-test for independent samples. Student's t-test tests the null hypothesis that the means of the two groups are equal, against the alternative hypothesis that the means are different. A significant result (*p*-value <α) leads to the rejection of the null hypothesis of equality and allows the inference of significant differences between the two samples. The assumption of data normality was validated (required for the application of parametric tests): the hypothesis of normality of the continuous variable in groups of less than 30 was not rejected, according to the Shapiro-Wilk test (*p*-value >0.05), and the shape of the approximate normal distribution in samples larger than 30 validated the application of Student's t-test. In addition, in samples of sufficiently large size (greater than 30) the Student's t-test is robust for small deviations from normality and the data distribution tends to Normal (by the Central Limit Theorem). A significance level of 5% (α=0.05) was considered.

## RESULTS

The COS cycles were stratified (n=246) according to the ORPI values of women submitted to ART. Almost half (48.4%) of the cycles (n=119) were performed in individuals in G1, rated as having low ovarian reserve and an ORPI <0.5; 46 cycles (18.7%) were perfomed in individuals in G2, rated as having normal ovarian reserve and an ORPI of [0.5-0.9]; and 81 cycles (32.9%) were performed in women in G3, rated as having high ovarian reserve and an ORPI ≥0.9.

Protocol 1 with rFSH alfa and beta (Gonal^®^/Benfola^®^/Puregon^®^) was prescribed in the majority of the COS cycles (82.5%, n=203), while the remaining 17.5% (n=43) were offered Protocol 2 with rFSH corifollitropin alfa (Elonva^®^). Patients on both protocols were prescribed uHMG (Menopur^®^) and offered rotection against early LH surge with a GnRH antagonist (Orgalutran^®^/Cetrotide^®^). Ovulation was triggered with rhCG (Ovitrelle^®^) in 87.8% of the cycles (n=216) and with a GnRH agonist (Decapeptyl^®^/Gonapeptyl^®^) in 11% of the cycles.

Protocol 1 was used in 68.1% (n=81) and Protocol 2 in 31.9% (n=38) of the women with low ovarian reserve (ORPI <5) assigned to G1. The average number of oocytes obtained was significantly higher in Protocol 1 (7.99±4.119) compared with Protocol 2 (4.50±3.585), t (117) = 4.228 and *p*-value <0.01; therefore, it is possible to reject the null hypothesis of equality of means between the two protocols. There is thus statistical evidence that the average number of oocytes obtained differs between the protocols, with an average difference of about three oocytes, with patients on Protocol 1 having, on average, three more oocytes than subjects on Protocol 2. The mean number of stimulation days was significantly higher in Protocol 2 (11.39±1.264) than in Protocol 1 (10.80±1.123), t (117) = -2.576 and *p*-value <0.05 with an average difference of 0.592 days. There is thus statistical evidence that the average number of stimulation days differs significantly between the two protocols.

**Figure 1:**
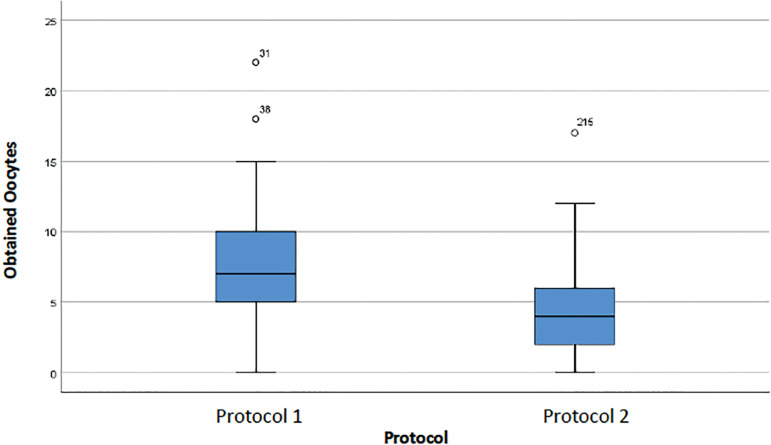
Oocytes retrieved from cycles performed in women with an ORPI <0.5 for each protocol.

The following boxplots (diagrams of extremes and quartiles) represent the distribution of data for each protocol regarding the number of oocytes obtained and the number of stimulation days. Figure 1 shows a greater variability in the number of oocytes obtained in Protocol 1; in Protocol 2, a smaller range of values is observed, and the median is smaller (horizontal line in the center of the box). The median number of oocytes in Protocol 1 was 7, while 50% of Protocol 2 patients had up to 4 oocytes (Median = 4). There are two outliers in Protocol 1 and one outlier in Protocol 2. Outliers are values that exceed 1.5 times the Interquartile Range (IQR = 3^rd^ Quartile-1^st^ Quartile). 

**Figure 2:**
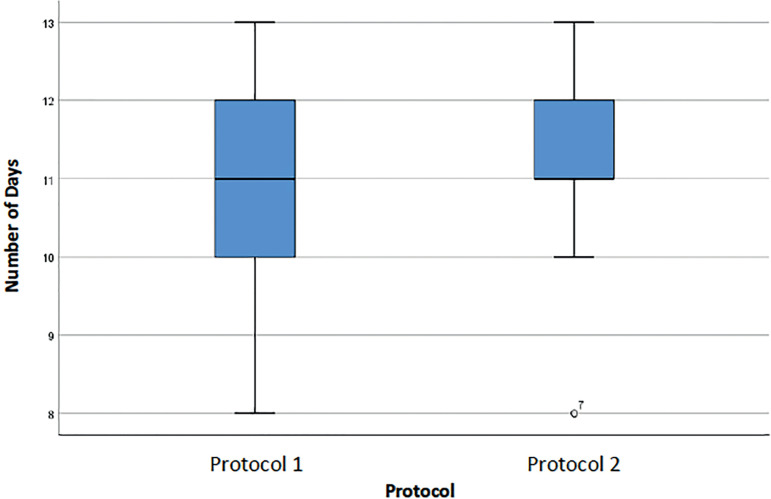
Number of days of stimulation in cycles of women with an ORPI <0.5 for each protocol.

Figure 2 describes the number of days of stimulation and shows that patients on Protocol 1 had a greater range of values (between 5 and 13 days), with a median of 11 days. The median was 11 days in the two Protocols, but the population was more homogeneous in Protocol 2 (half of the patients had 10-11 days of stimulation, while in Protocol 1 half of the subjects had 5-11 days of stimulation). 

The descriptive statistics findings about the medication dose for each protocol prescribed to women in with an ORPI <0.5 in G1 revealed that in Protocol 1, the dose of rFSH alfa and beta varied between 1125 and 3000 International Units (IU), with an average value of 2121.60±493.896 IU. The uHMG dosage oscillated between 375 IU (minimum) and 1800 IU (maximum) with an average value of 935.19±290.737 IU. The GnRH antagonist dose varied in this group between 0.25 mg and 2 mg, with an average value of 1.28±0.301 mg. The patients in the 38 cycles performed on Protocol 2 were prescribed 150 µg of rFSH corifollitropin alfa. The doses of uHMG oscillated between 300 IU (minimum) and 3900 IU (maximum), with an average value of 1851.32±02.937 IU. The GnRH antagonist dose varied between 0.75mg and 2.25 mg, with an average value of 1.520±0.331 mg.

In general, we observed that the average doses of uHMG and GnRH antagonists were higher in Protocol 2 than in Protocol 1. The comparison of the proportion of mature oocytes (mean number of mature oocytes / mean number of oocytes obtained) using different triggers is shown in Tables 1 and 2.

**Table 1.** Classification of the cycles of women with an ORPI <0.5 based on the trigger.

		Frequency	Percentage	Valid Percentage	Cumulative Percentage
Valid	rhCG (Ovitrelle^®^)	113	95.0	97.4	97.4
	GnRH agonist (Decapeptyl^®^/Gonapeptyl^®^)	3	2.5	2.6	100.0
	Total	116	97.5	100.0	
Missing	System	3	2.5		
Total	119	100.0			

**Table 2.** Student’s t-test comparing the proportion of mature oocytes with the trigger, in the cycles of women with an ORPI <0.5.

		n	Average	Standard deviation	t (df)	*p*-value	Mean difference	95% Confidence Interval	
								Inferior limit	Superior limit
Mature oocytes	rhCG (Ovitrelle^®^)	113	0.7759	0.24219	-0.830 (144)	0.408	-0.117	-0.39558	0.16191
	GnRH agonist (Decapeptyl^®^/Gonapeptyl^®^)	3	0.8928	0.11625					

In this group, there was no evidence of a significant difference in the proportion (percentage) of mature oocytes for different triggers (*p*-value >0.05). The values did not differ significantly, although in the group given GnRH agonists there were approximately 12% more mature oocytes than in the group given rhCG.

Protocol 1 was prescribed to 93.5% (n=43) and Protocol 2 to 6.5% (n=3) of the women with normal ovarian reserve (ORPI [0.5-0.9]) in G2. As for the average number of oocytes obtained, there was no statistical evidence to reject the null hypothesis of equality between the two protocols, t (44) = 0.559, *p*-value =0.579. The average number of oocytes obtained with Protocol 1 (10.91±4.710 oocytes) and with Protocol 2 (9.33±4.726 oocytes) did not differ significantly. The number of stimulation days was significantly higher in Protocol 2, t (44) = - 2.130, *p*-value <0.05, with an average difference of 1.6 days. Patients on Protocol 2 had an average of 12±1.00 days of stimulation, while subjects in Protocol 1 had an average of 10.37±1.291 days of stimulation.

**Figure 3:**
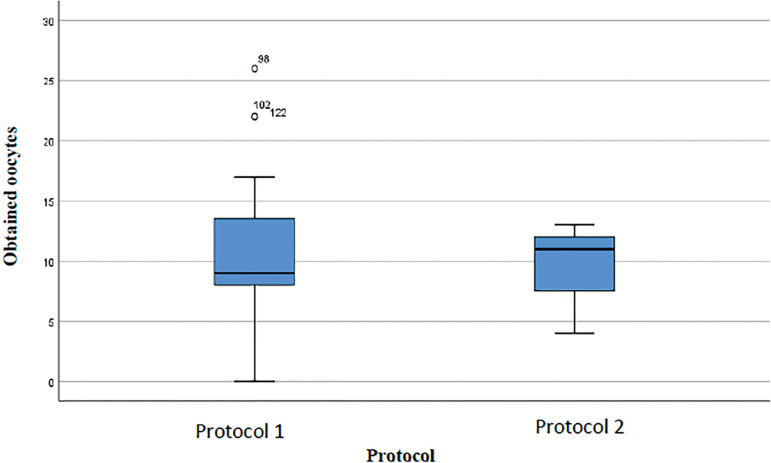
Oocytes retrieved from the cycles of women with an ORPI [0.5-0.9] for each protocol.

**Figure 4:**
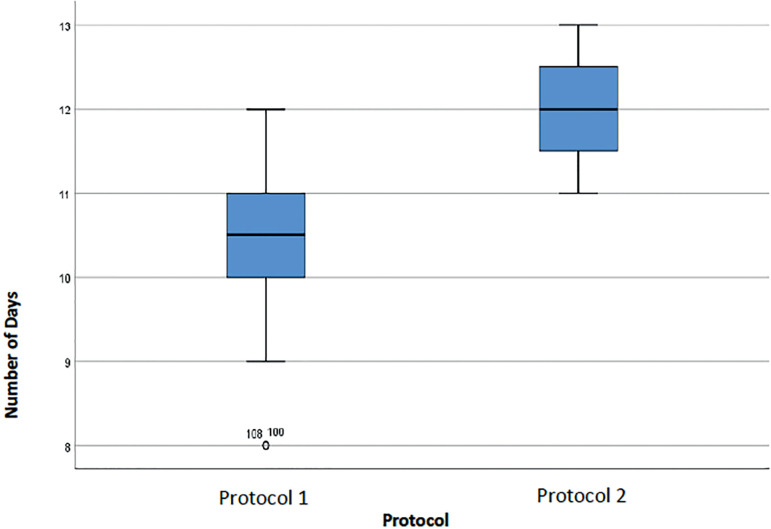
Number of days of stimulation in the cycles of women with an ORPI [0.5-0.9] for each protocol.

Figure 3 shows the median number of oocytes obtained from the two protocols (9 in Protocol 1 and 11 in Protocol 2). As for the number of days of stimulation (Figure 4), subjects on Protocol 2 were on stimulation for a median of 12 days (50% of the population were on stimulation for up to 12 days), while patients on Protocol 1 were on stimulation for a median of 10.5 days.

The dose of rFSH alfa and beta prescribed to patients on Protocol 1 ranged from 900 IU and 2700 IU, with an average value of 1777.91±479.186 IU. The dose of uHMG oscillated between 375 IU and 1800 IU, with an average value of 856.40±305.850 IU. The dose of GnRH antagonists varied in this group between 0.75 mg and 1.75 mg, with an average value of 1.2209±0.31908 mg. The three cycles performed in patients on Protocol 2 used a dose of 150µg of rFSH corifollitropin alfa. The dose of uHMG oscillated between 1800IU and 2700IU, with an average value of 2200±458.258 IU. The dose of GnRH antagonists varied between 1 mg and 2 mg, with an average value of 1.6667±0.57735 mg. In general, we observed that the average doses of uHMG and GnRH antagonists were higher in Protocol 2 than in Protocol 1. The comparison of the proportion of mature oocytes (mean number of mature oocytes / mean number of oocytes obtained) using different triggers is shown in Tables 3 and 4.

**Table 3.** Classification of the cycles of women with an ORPI of [0.5-0.9] based on the trigger.

		Frequency	Percentage	Valid Percentage	Cumulative Percentage
Valid	rhCG (Ovitrelle^®^)	41	89.1	89.1	89.1
	GnRH agonist (Decapeptyl^®^/Gonapeptyl^®^)	5	10.9	10.9	100.0
	Total	46	100.0	100.0	

**Table 4.** Student’s t-test comparing the proportion of mature oocytes with the trigger, in the cycles of women with an ORPI of [0.5-0.9].

		n	Average	Standard deviation	t (df)	*p*-value	Mean difference	95% Confidence interval	
								Inferior limit	Superior limit
Mature oocytes	rhCG (Ovitrelle^®^)	41	0.7905	0.18711	-0.119 (44)	0.906	0.001	-0.18562	0.16486
	GnRH agonist (Decapepty^®^/Gonapeptyl^®^)	5	0.8009	0.14338					

In this group, there was no statistical evidence to reject the null hypothesis of equality of the percentage of mature oocytes with each trigger (*p*-value >0.05). The values were similar, and the difference, a residual 1%.

**Figure 5:**
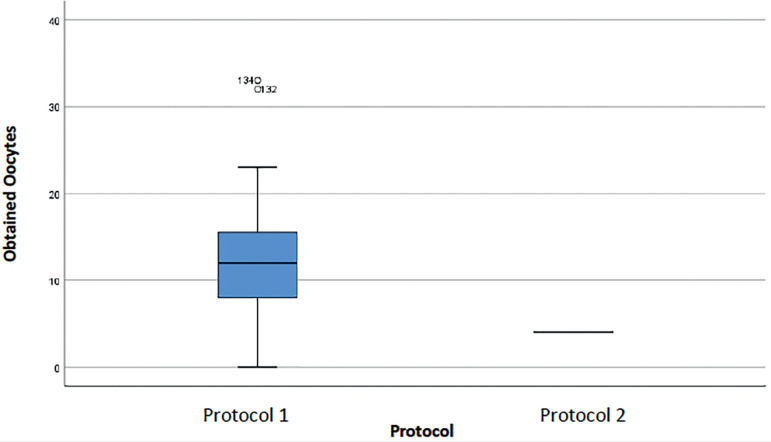
Oocytes retrieved from the cycles of women with an ORPI ≥ 0.9 for each protocol.

Protocol 1 was prescribed to 97.5% (n=79) and Protocol 2 to 2.5% (n=2) of the women with high ovarian reserve (ORPI ≥ 0.9) in G3. In this group, the number of oocytes obtained did not differ significantly between protocols: in Protocol 1, the average was 12.18±6.207 oocytes; in Protocol 2, it was 4.00±0.00 oocytes (zero variability, as seen in the boxplot: the two subjects in this condition obtained 4 oocytes), with a *p*-value of 0.068. Regarding the average number of days of stimulation of the two protocols, there was no statistical evidence to reject the null hypothesis of equality (*p*-value >0.05). The following boxplots represent the distribution of data for each protocol regarding the number of oocytes obtained and the number of stimulation days. In Figure 5, despite the higher median number of obtained oocytes in Protocol 1, we could not infer a trend for the study population (*p*-value >0.05).

**Figure 6:**
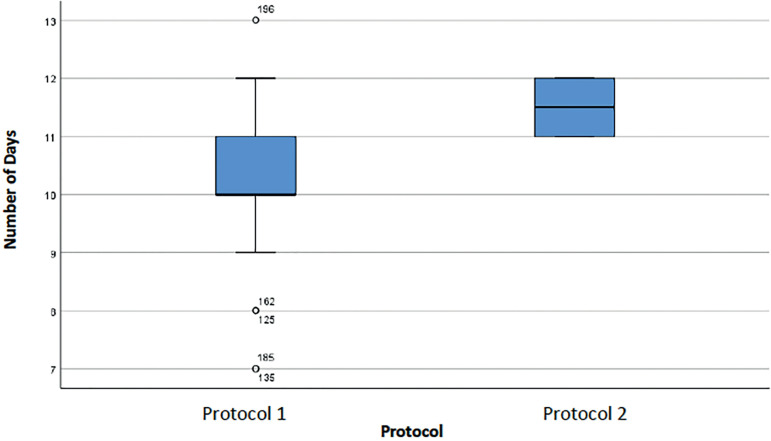
Number of days of stimulation in the cycles of women with an ORPI ≥ 0.9 for each protocol.

Although the median length of stimulation was higher in Protocol 2 (Figure 6), the difference in relation to Protocol 1 was not statistically significant. In terms of the dosages for each medication prescribed, patients on Protocol 1 were prescribed between 950 and 2475 IU of rFSH alfa and beta, with an average value of 1411.01±322.298 IU. The dose of uHMG oscillated between 225 IU (minimum) and 1425 IU (maximum), with an average value of 652.78±236.326 IU. The dose of GnRH antagonists varied in this group between 0.50 mg and 1.75 mg, with an average value of 1.1930 ± 0.24334 mg. The dose of 150 µg of rFSH corifollitropin alfa was used in the two cycles performed with patients on Protocol 2. The dose of uHMG oscillated between 1800 IU (minimum) and 2100 IU (maximum), with an average value of 1950±212.132 IU. The dose of GnRH antagonists varied between 1.50 mg and 1.75 mg, with an average value of 1.6250±0.17678 mg. In general, we observed that the average doses of uHMG and GnRH antagonists were higher in Protocol 2 than in Protocol 1. The comparison of the proportion of mature oocytes (mean number of mature oocytes / mean number of oocytes obtained) using different triggers is shown in Tables 5 and 6. In this group, there was no statistical evidence to reject the null hypothesis of equality in the percentage of mature oocytes with the different triggers (*p*-value >0.05). The values were similar, and the average difference, a residual 2%.

**Table 5.** Classification of the cycles of women with an ORPI ≥ 0.9 based on the trigger.

		Frequency	Percentage	Valid Percentage	Cumulative Percentagem
Valid	rhCG (Ovitrelle^®^)	62	76.5	76.5	76.5
	GnRH agonist (Decapeptyl^®^/Gonapeptyl^®^)	19	23.5	23.5	100.0
	Total	81	100.0	100.0	

**Table 6.** Student’s t-test comparing the proportion of mature oocytes with the trigger, in the cycles of women with an ORPI ≥ 0.9.

		n	Average	Standard deviation	t (df)	*p*-value	Mean difference	95% confidence interval	
								Inferior limit	Superior limit
Mature oocytes	rhCG (Ovitrelle^®^)	62	0.8109	0.17670	0.472 (79)	0.638	0.021	-0.06863	0.11131
	GnRH agonist (Decapeptyl^®^/Gonapeptyl^®^)	19	0.7895	0.15686					

## DISCUSSION

Similarly to reports from other countries, this study found that Portuguese patients seek ART therapies at a more advanced age, as evinced by the mean age of the individuals included in our study population (34.51 years). A significant portion of study participants was described as having low ovarian reserve (48.4%). Social, biological, and public health factors contribute to this significant increase in infertility cases. Knowledge about the management of follicular stimulantion agents (uHMG and rFSH) has increased the confidence in the protocols prescribed in this study, resulting in prescription to a greater number of patients (Protocol 1 in 82.5% and Protocol 2 in 17.5%). In the last five years, the advent of a new method to protect patients against spontaneous LH surges coupled with better control of COS has allowed for better pregnancy rates and the possibility of avoiding severe OHSS, one of the most frequent and feared complications of IVF/ICSI procedures. According to the 2020 Guidelines of the European Society of Human Reproduction and Embryology (ESHRE) (Ovarian Stimulation TEGGO *et al*., 2020), the use of protocols with GnRH antagonists is recommended over the use of protocols with GnRH agonists, given the comparable effectiveness, but greater safety of GnRH antagonists. In both protocols under study, the association of rFSH with uHMG (FSH + LH) was used, due to the possibility of greater sensitization of oocyte FSH receptors when exposed to LH and circulating endogenous estrogen. However, there is still controversy regarding this association. Some studies reported no benefit in combining rFSH with uHMG, compared with rFSH alone, in protocols in which GnRH antagonists were used (Martin-Johnston *et al*., 2007). Other studies reported that the association led to an increase in the proportion of oocytes obtained, when compared with the use of rFSH or uHMG alone (Pacchiarotti *et al*., 2016).

Other studies have reported that patients on pituitary suppression induced by GnRH analogs (either agonists or antagonists) may experience profound LH suppression, resulting in lower fertilization rates and increased risk of miscarriage. These patients require high doses of FSH and have better implantation rates with the addition of LH to the FSH stimulation protocol (Fleming *et al*., 1998).

The difference between the protocols under analysis lies in the way the prescribed gonadotropins were used. In Protocol 1, rFSH alfa or beta was used in daily doses; in Protocol 2, a deposit form with prolonged release rFSH (corifollitropin alfa) was used. The maximum daily dose of rFSH alfa or beta has been widely studied and should not exceed 300 IU daily. The total dose used to be defined based on the number of days required to reach oocyte maturation. Regarding corifollitropin alfa, even though the maximum dose is inversely proportional to body weight (100µg in women <60 kg and 150µg in women > 60 kg) to protect against OHSS, in patients with low reserve or poor response to prior COS (<6 oocytes), the maximum dose (150µg) is recommended in order to produce better ovarian response, regardless of body weight.

In the Reproductive Medicine Unit of CHUCB, in addition to the pre-determined COS protocols, COCs are also used to schedule and date patient menstruation, so as to facilitate scheduling of cycles and minimize the operational costs of IVF/ICSI procedures by optimizing the use of growth medium, equipment, and working time. According to studies (Biljan *et al*., 1998; Griesinger *et al*., 2008), the use of COC offers other advantages, such as improving follicular synchronism during ovulation stimulation, decreasing cancellation rates in hyper-responsive women, and yielding a greater number of oocytes. However, according to the ESHRE guidelines (Ovarian Stimulation TEGGO *et al*., 2020) mentioned above, the use of COC before COS is not recommended in protocols in which GnRH antagonists are used since they may decrease treatment efficacy, as evidence indicates that pregnancy rates are lower pre-treatment with COC is performed. However, it is difficult to assess the interference of pre-treatment with COC on outcomes of full-term pregnancy, since in a COS cycle there are numerous steps that may interfere with the final outcome. Regarding the number of oocytes retrieved from OPU, numbers were higher when recombinant follitropin alfa and beta were used in the three study groups compared with deposited corifollitropin alfa.

This difference was significant (*p*-value <0.001) only in the group of women with a low ovarian reserve of an ORPI <0.5. Other studies have found different results. A clinical trial published in 2015 (Kolibianakis *et al*., 2015) showed that, in a certain group of women with a predictable weak response to COS, there was no significant difference between the use of the different types of rFSH. The ESHRE guidelines (Ovarian Stimulation TEGGO *et al*., 2020) state that the use of daily rFSH or long-acting rFSH is also recommended in cycles in which GnRH antagonists are used in women expected to have good response to COS. Further studies are certainly needed. The length of stimulation required compared with the ten-day pattern of predicted follicular evolution was significantly higher (*p*-value <0.05) with the use of corifollitropine alfa in two of the groups (G1 and G2). Length of stimulation was added by one day in the low reserve group and two days in the normal reserve group. The need for complementation with HMG in greater quantities is inherent to this protocol. Regarding the number of hormones administered necessary for COS, it is possible to verify that the total dose of rFSH alfa or beta / urinary HMG used was inversely proportional to the patient's ovarian reserve. In other words, the lower the ORPI value, the higher the average number of IU administered (Oliani *et al*., 2019; Oliveira *et al*., 2012). Thus, it is possible to understand the importance of knowing the potential ovarian response of a woman in the individualization of the medication dose, so as to try and avoid either a weak response to treatment or hyperstimulation, which may lead to OHSS.

We could also verify that there was a need for complementation with uHMG in higher doses when corifollitropin alfa was used as an rFSH agent. The dosage of the GnRH antagonist is dependent on the number of COS days until the trigger is used, since the antagonist's goal is to prevent a premature LH surge and thus prevent the loss of the cycle resulting from early ovulation. The higher mean dose of antagonists required with the use of corifollitropin alfa relates to the greater number of days of stimulation required with this protocol. In both protocols, daily doses of 0.25 mg were used, according to a flexible protocol (in which the antagonist is introduced when at least one follicle with> 14 mm is seen in the control ultrasound) (Tarlatzis *et al*., 2006; Al-Inany *et al*., 2007). Regarding the trigger used, there was no significant difference between the use of rhCG or GnRH agonists in obtaining mature oocytes. The percentages of mature oocytes were similar in the three ORPI groups and varied between 78% and 89% in terms of effectiveness, regardless of the trigger used. The ESHRE guideline (Ovarian Stimulation TEGGO *et al*., 2020) also shows that, in women with predictable poor response to treatment (poor responders and low reserve) and predictable good response (normal reserve), only uhCG or rhCG should be used as a trigger, while GnRH agonists should be avoided. The latter should be reserved for the final maturation of oocytes in women at risk of hyperstimulation (increased reserve), since it has been associated with a lower risk of OHSS compared with hCG.

The patients in this study prescribed GnRH agonists to trigger ovulation did not have embryos transferred, since they were cryopreserved for embryo transfer (CET) in a natural cycle. With GnRH agonists, the total amount of gonadotropins released to support the formation of the corpus luteum is not adequate, which thus leads to premature luteolysis and poor reproductive results (Yılmaz *et al*., 2020). Thus, the use of a GnRH agonist for final maturation and fresh transfer is not recommended in the general IVF / ICSI population (Ovarian Stimulation TEGGO *et al*., 2020). With the increase in the effectiveness of cryopreservation, the use of GnRH agonists with a "freeze all" approach so that embryos are transferred in subsequent cycles may be the ideal strategy to decrease or eliminate the prevalence of OHSS (Yılmaz *et al*., 2020). The main limitations of this study are the sample size and the number of COS protocols compared. In the future, we believe this study should be extended to cover a larger population, compare between other COS protocols, and use data from other Reproductive Medicine Units in the country, so that a more thorough analysis is performed.

## CONCLUSION

From the available data, it is possible to conclude that follitropin alfa or beta (Protocol 1) yielded a greater number of oocytes retrieved in OPU than corifollitropin alfa (Protocol 2) for all ORPI ranges, with a significant difference (*p*<0.001) seen in the low reserve group (G1); the dose of rFSH alfa or beta used in conjunction with uHMG (Protocol 1) increases in an inversely proportional manner to the ORPI value; the fixed dose of rFSH deposit used in Protocol 2 requires a sharp increase in the dose of uHMG, also reflected in the number of COS days and, consequently, in the number of ampoules used by the GnRH antagonist; and the final maturation (trigger) to obtain mature oocytes (MII) has the same efficiency with the use of rhCG or a GnRH agonist. Further studies, using ORPI as an additional input for the selection of users directed to ART procedures, are necessary for the implementation of other COS protocols.
